# Retalho reverso de artéria interóssea posterior: Indicações, técnica e complicações em 18 casos consecutivos

**DOI:** 10.1055/s-0045-1812468

**Published:** 2025-12-10

**Authors:** Alvaro Baik Cho, Antonio Isidoro de Sousa Neto, Kríssia Caroline Soares Franco, Marcelo Rosa de Rezende, Teng Hsiang Wei, Rames Mattar Junior

**Affiliations:** 1 Grupo de Cirurgia da Mão e Microcirurgia Reconstrutiva, Instituto de Ortopedia e Traumatologia, Hospital das Clínicas da Faculdade de Medicina da Universidade de São Paulo, São Paulo, SP, Brasil; 2 Departamento de Ortopedia e Traumatologia, Faculdade de Medicina da Universidade de São Paulo, São Paulo, SP, Brasil

**Keywords:** fáscia/transplante, lesões dos tecidos moles, retalhos cirúrgicos, traumatismos da mão, fascia/transplantation, hand injuries, soft tissue injuries, surgical flaps

## Abstract

**Objetivo:**

Avaliar a taxa de sucesso do retalho reverso da artéria interóssea posterior e inferir sua previsibilidade, confiabilidade e segurança.

**Métodos:**

Conduzimos um estudo retrospectivo observacional de 18 pacientes com lesões de partes moles no terço distal do membro superior submetidos a retalho da artéria interóssea posterior. Foram analisados o tamanho do retalho, a área receptora, a necessidade de enxertia complementar, o resultado clínico e as complicações.

**Resultados:**

Houve uma perda total e uma parcial, o que resultou em taxa de sucesso de 94,45%. A maioria dos casos apresentou boa cicatrização e ausência de complicações importantes. Foi necessária enxertia em 55,5% dos casos. Em quatro casos, o retalho mostrou-se insuficiente para a cobertura da falha, sendo necessário enxerto cutâneo em áreas de granulação. Em quatro casos, foi realizada cobertura de polegar, que é fundamental para a funcionalidade da mão. A média do tamanho foi de 9,6 cm de comprimento por 4,4 cm de largura, com variação de comprimento de 5,5 a 13 cm e de largura, de 3 a 7 cm. O retalho demonstrou utilidade em áreas funcionais como o polegar e a primeira comissura.

**Conclusão:**

O retalho da artéria interóssea posterior é uma opção segura e eficaz para a cobertura de falhas no terço distal do membro superior, especialmente no dorso da mão e no polegar, com baixa morbidade e bons resultados funcionais e estéticos.

**Nível de Evidência:**

IV, série de casos.

## Abstract (en)

**Objective:**

To evaluate the success rate of the reverse posterior interosseous artery flap, inferring its predictability, reliability, and safety.

**Methods:**

We conducted a retrospective observational study including 18 patients with soft tissue lesions in the distal third of the upper limb who underwent posterior interosseous artery flap surgery. The study analyzed the flap size, the recipient area, the need for additional grafting, the clinical outcome, and the complications.

**Results:**

There was one total and one partial loss of the flap, resulting in a success rate of 94.45%. Most cases healed well, and we did not observe major complications. More than half (55.5%) of the cases required grafting. In four cases, the flap was insufficient to cover the defect, requiring skin grafting in areas of granulation. In four cases, we performed thumb coverage, which is essential for hand function. The average flap size was of 9.6 cm in length by 4.4 cm in width, with the length ranging from 5.5 to 13 cm, and the width, from 3 to 7 cm. The flap was beneficial in functional areas such as the thumb and first commissure.

**Conclusion:**

The posterior interosseous artery flap is a safe and effective option to cover defects in the distal third of the upper limb, especially on the dorsum of the hand and thumb, with low morbidity and good functional and esthetic outcomes.

**Level of Evidence:**

IV, case series.

## Introdução

Defeitos nos tecidos moles da mão são lesões desafiadoras, pois frequentemente são associados à exposição de tendões ou estruturas neurovasculares, 1 e requerem uma cobertura cutânea adequada para uma reabilitação precoce, para prevenir rigidez e contraturas.

A literatura descreve diversas opções de coberturas, sejam retalhos locais, pediculados ou microcirúrgicos. A escolha do retalho é baseada em grande parte na preferência e conforto do cirurgião, e leva em consideração fatores como grau de dificuldade técnica, comprimento do pedículo, volume e potencial para fechamento primário.

A reconstrução desses defeitos em estágio único permite mobilização precoce, reduz a permanência hospitalar, minimiza a infecção e, assim, alcança-se um bom resultado funcional. Retalhos regionais compartilham dessas vantagens, pois reduzem o tempo cirúrgico e apresentam menor dificuldade técnica quando comparados com retalhos microcirúrgicos. 2

Os retalhos regionais mais utilizados nesses casos são os fasciocutâneos antebraquiais de fluxo retrógrado da artéria radial, da artéria ulnar e da artéria interóssea posterior (AIP). Os dois primeiros apresentam a desvantagem de sacrificarem artérias de maior importância para a mão, com associação de maior morbidade da área doadora. Outra desvantagem e limitação desses retalhos é a necessidade de o arco palmar estar preservado para assegurar a sua perfusão. Não é incomum que o arco esteja comprometido nas lesões mais graves da mão.

O retalho da AIP foi publicado originalmente de forma independente por dois grupos para cobertura de defeitos no cotovelo. 3 4 5 Posteriormente, passou a ser utilizado em defeitos distais após sua descrição para fluxo retrógrado, 6 7 com base em sua anastomose com a AIP, e apresenta-se como interessante opção para coberturas pela sua confiabilidade e previsibilidade, 8 especialmente na região dorsal da mão até as articulações interfalangeanas proximais e a região da primeira comissura. 9

Este estudo tem como objetivo avaliar a taxa de sucesso do retalho da AIP e inferir sua previsibilidade, confiabilidade, e o grau de segurança da realização do retalho.

## Métodos

Este estudo foi aprovado pelo Comitê de Ética institucional, conforme parecer n° 7.362.990/2025 (CAAE: 85725225.2.0000.0068).

Trata-se de estudo retrospectivo primário, observacional e descritivo de uma série de 18 casos consecutivos de pacientes com perdas de cobertura cutânea no terço distal do punho, da mão e da primeira comissura, secundárias a traumas ou infecções. Os pacientes foram operados de setembro de 2001 a junho de 2023, e todas as cirurgias foram realizadas pelo mesmo cirurgião, especialista em mão e microcirurgia.

O retalho foi indicado para cobertura por amputação no nível do carpo em três casos, por amputação no nível do primeiro metacarpo em um caso, por amputação no nível da articulação interfalangeana do polegar em um caso, por falha de cobertura na região dorsal da mão em cinco casos, por falha de cobertura na região dorsal, no nível do primeiro metacarpo, em três casos, por falha no dorso radial, no punho, em um caso, por falha na região volar radial do punho em um caso, para cobertura da primeira comissura em um caso, por retração cicatricial da primeira comissura em um caso, e por falha de cobertura na região tenar em um caso.

Foram avaliados dados como as dimensões da falha e do retalho, os locais receptores e a gravidade das lesões, a necessidade de enxertia de pele nas áreas doadora e receptora, e as complicações (infecção e perda parcial ou total). Pacientes submetidos a outras opções de cobertura, sejam retalhos regionais ou microcirúrgicos, foram excluídos do trabalho.

## Técnica Cirúrgica

Iniciou-se com as marcações anatômicas: linha traçada do epicôndilo lateral do úmero distalmente à articulação radioulnar distal (ARUD). Depois, foi marcado um ponto 2 cm próximo à ARUD, a provável localização da anastomose entre a AIP e a artéria interóssea anterior, como ponto de rotação do pedículo do retalho.

**Fig. 1:**
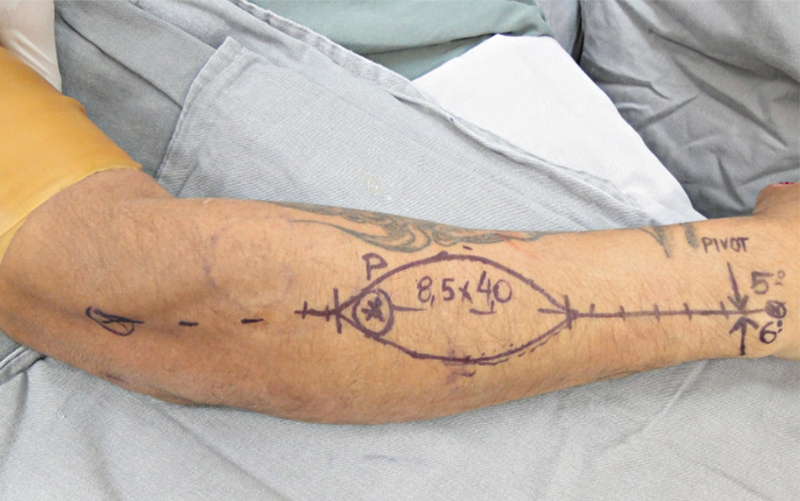
Marcações anatômicas.

**Fig. 2:**
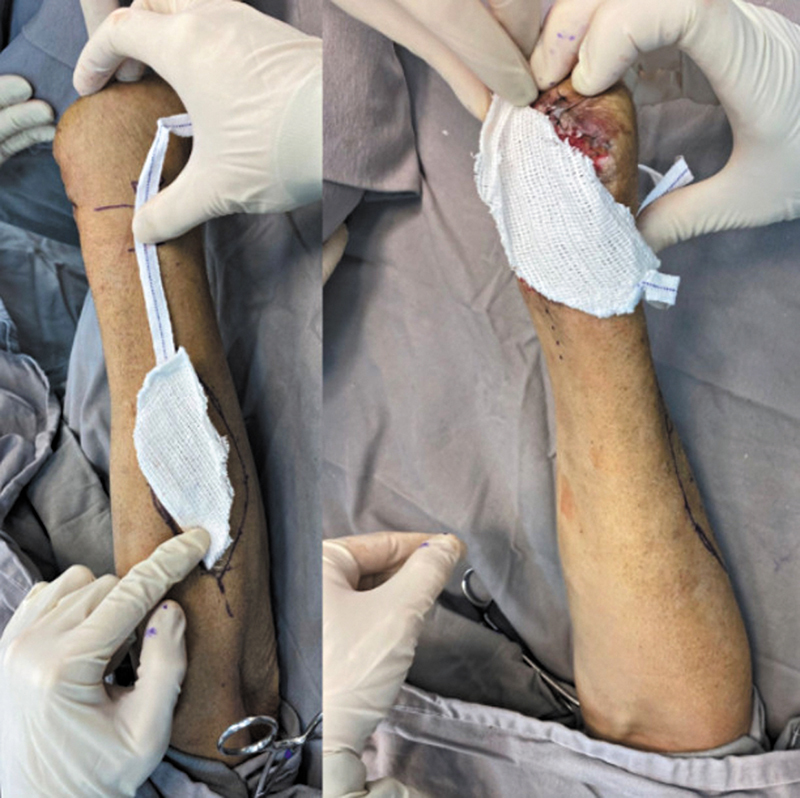
Modelo para a rotação do retalho.

Em seguida, dividiu-se a linha original em três partes iguais, com o terço médio representando a localização ideal para a retirada do retalho fasciocutâneo, onde fica localizada a sua perfurante mais relevante ( Fig. 1 ). Depois, realizou-se o desbridamento do defeito de cobertura e a medição da distância do ponto de rotação do retalho até o defeito, medida esta que indicou o comprimento do retalho e do pedículo a ser dissecado ( Fig. 2 ).

**Fig. 3:**
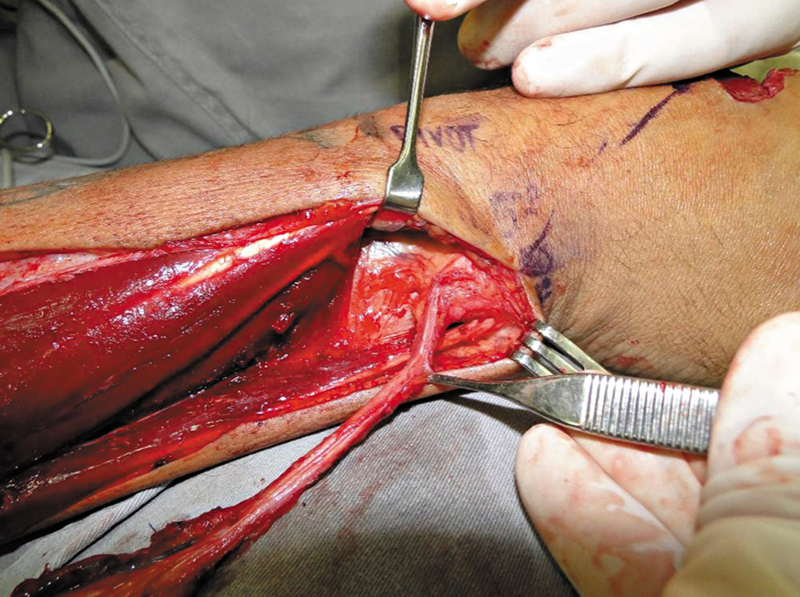
Pedículo dissecado até o ponto de rotação.

**Fig. 4:**
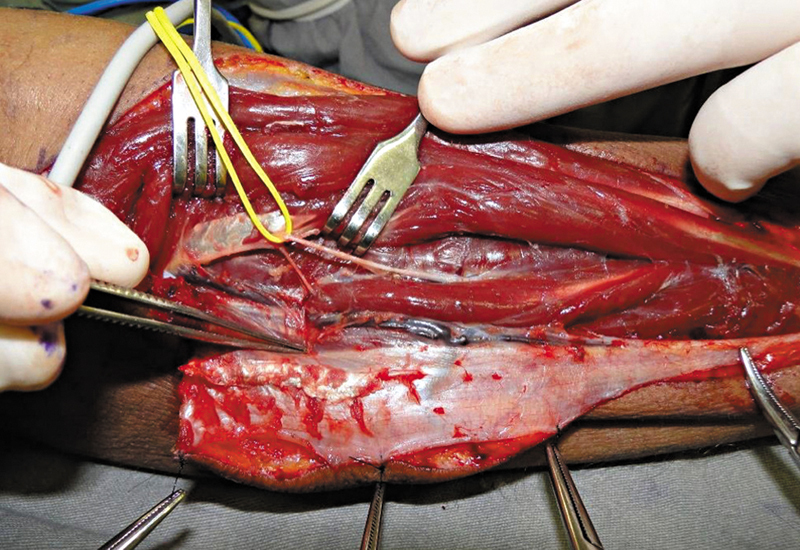
Nervo interósseo posterior separado do pedículo.

A dissecção sempre foi iniciada pela incisão distal e pela identificação dos tendões do extensor ulnar do carpo, no sexto compartimento extensor, e do extensor do dedo mínimo, no quinto compartimento. O pedículo da AIP foi encontrado entre os tendões. seguirem seguida, a fáscia foi incisada longitudinalmente sobre esses tendões, que foam retraídos cuidadosamente para a identificação e a confirmação da presença da artéria. Em todos os casos, a artéria situava-se mais próxima do tendão do quinto compartimento. Então, a dissecção prosseguiu de distal para proximal, até o ponto de rotação ( Fig. 3 ). O nervo interósseo posterior foi cuidadosamente dissecado da artéria ( Fig. 4 ). Em nenhum caso um ramo do nervo precisou ser seccionado.

**Fig. 5:**
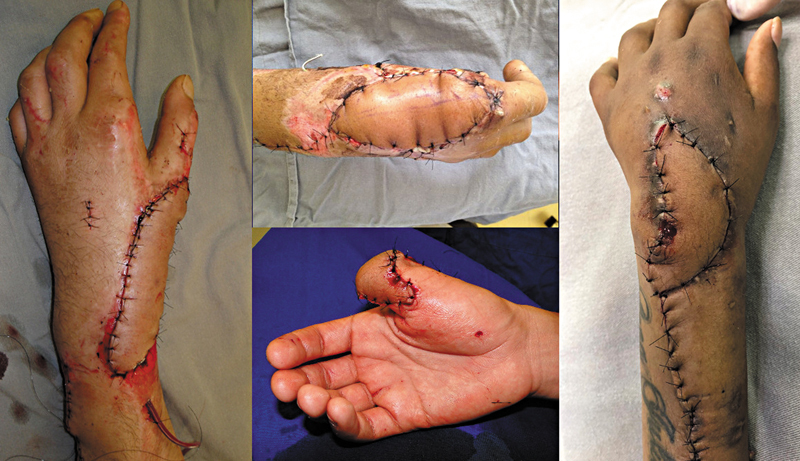
Falhas cobertas pelo retalho.

Em todos os casos, o retalho foi suficiente para a cobertura de estruturas nobres expostas ( Fig. 5 ), mas em alguns casos foi necessário complemento com enxerto parcial de pele em áreas marginais, associado ao retalho para cobertura total da lesão. Quando possível, foi realizado fechamento primário de área doadora. Se necessário, a área doadora foi fechada com enxerto parcial de pele. Em 12 casos, o retalho foi passado por túnel subcutâneo e, em 6 casos, com incisão.

Após procedimento cirúrgico, foram verificadas regularmente as condições do retalho, por meio de parâmetros como coloração, sangramento e tempo de enchimento capilar. Os pacientes ficaram com imobilização antebraquiopalmar por 2 semanas para a autonomização do retalho, e, depois, realizou-se acompanhamento mensal de funcionalidade, com seguimento variável.

## Resultados

Foram realizados 18 procedimentos cirúrgicos. Ao todo, 17 falhas de cobertura ocorreram por eventos traumáticos, e 1 falha foi atribuída à pioartrite do punho ( Tabela 1 ). O sexo masculino foi predominante, assim como a ocorrência de lesões na mão direita, com 11 casos afetando a mão direita e 7, a mão esquerda.

**Tabela 1:** Aspectos descritivos dos casos operados com retalho da artéria interóssea posterior em membros superiores

Casos ( *n* = 18)	Sexo	Local da falha de cobertura*	Tamanho (cm)**	Fechamento da área doadora	Enxerto na área receptora***	Desfecho	Etiologia
1	Masculino	Amputação do primeiro metacarpo	5,5 × 4,0	Primário	−	Favorável	Trauma
**2**	Masculino	Amputação no nível do carpo	8,0 × 4,5	Enxertia	−	Favorável	Trauma
**3**	Masculino	Dorso da mão	6,5 × 4,0	Primário	−	Favorável	Pioartrite
**4**	Masculino	Dorso do primeiro metacarpo	9,0 × 5,0	Enxertia	−	Favorável	Trauma
**5**	Masculino	Dorso radial do punho e da mão	12 × 4,5	Enxertia	−	Favorável	Trauma
**6**	Masculino	Região tenar	9,0 × 4,0	Enxertia	Sim	Favorável	Trauma
**7**	Masculino	Dorso da mão	12,0 × 7,0	Enxertia	−	**Perda parcial**	Trauma
**8**	Masculino	Polegar no, nível da articulação interfalangeana	9,0 × 4,0	Primário	−	Favorável	Trauma
**9**	Masculino	Amputação no nível do carpo	13,0 × 4,5	Enxertia	Sim	Favorável	Trauma
**10**	Masculino	Amputação no nível do carpo	13,0 × 4,5	Enxertia	Sim	Favorável	Trauma
**11**	Masculino	Dorso radial da mão	11,0 × 5,0	Enxertia	−	Favorável	Trauma
**12**	Masculino	Dorso do primeiro metacarpo	13,0 × 5,5	Primário	−	Favorável	Trauma
**13**	Masculino	Dorso do primeiro metacarpo	8,5 × 4,0	Primário	−	Favorável	Trauma
**14**	Masculino	Dorso da mão	7,5 × 4,0	Enxertia	−	**Perda total**	Trauma
**15**	Feminino	Dorso radial do punho	7,0 × 3,0	Primário	−	Favorável	Trauma
**16**	Masculino	Regiões volar e radial do punho	12,5 × 5,0	Primário	−	Favorável	Trauma
**17**	Masculino	Primeira comissura	5,5 × 4,0	Enxertia	−	Favorável	Trauma
**18**	Feminino	Primeira comissura	11,0 × 3,0	Primário	−	Favorável	Trauma

Notas:^*^ Indicação ou local da falha de cobertura; ^**^ retalho em centímetros (comprimento x largura); e ^***^ enxertia complementar ao retalho na área receptora.

Em relação às dimensões dos retalhos, o maior apresentou 13 × 5,5 cm, e o menor, 7 × 3 cm, com uma média de 9,6 × 4,4 cm. Desse modo, o comprimento dos retalhos variou de 5,5 cm a 13 cm, e a largura, de 3 cm a 7 cm ( Tabela 1 ).

**Fig. 6:**
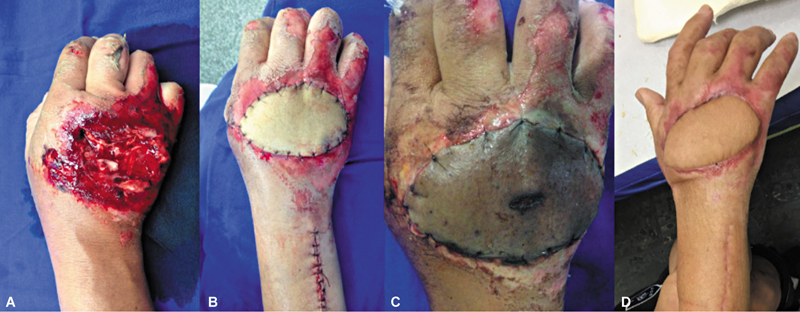
( **A** ) Falha inicial; ( **B** ) pós-operatório imediato de retalho da artéria interóssea posterior; ( **C** ) necrose total do retalho; e ( **D** ) pós-operatório tardio de retalho da artéria radial em paciente com flexoextensão de punhos e dedos preservados.

Em termos de desfecho, 1 caso (5,5%) evoluiu com perda total do retalho, e outro (5,55%), com perda parcial. O caso de perda total ocorreu em um paciente com falha de cobertura no dorso da mão devido a trauma, sendo realizada a remoção da área necrótica do retalho e a confecção de um retalho antebraquial pediculado de artéria radial, com resultado satisfatório ( Fig. 6 ). O caso de perda parcial também envolveu falha de cobertura no dorso da mão por trauma, com necrose parcial periférica das bordas distal e radial do retalho, após epidermólise. Optou-se por acompanhamento com cicatrização por segunda intenção. Em termos de eficácia na cobertura da área exposta, somente o retalho com perda total não cumpriu sua função, o que resultou em uma taxa de sucesso de 94,45%.

**Fig. 7:**
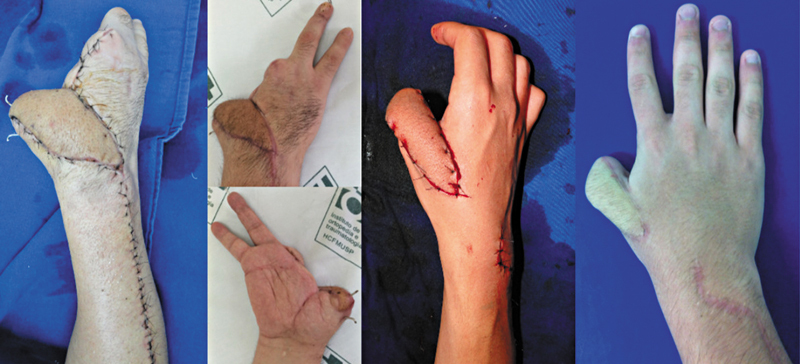
Casos de retalho para o polegar.

A função da mão ou do punho não foi objeto de estudo nesta pesquisa, pois depende de variáveis alheias ao uso dos retalhos, especialmente a gravidade inicial do trauma. Contudo, em casos específicos, o retalho demonstrou-se eficaz na manutenção da função do membro, particularmente em casos graves de amputação parcial do polegar, nos quais o retalho possibilitou a preservação do maior comprimento possível do dedo e de movimentos como a pinça e a oponência, em 4 dos 18 casos ( Fig. 7 ). De forma geral, 6 (33,33%) casos foram destinados à cobertura de regiões do polegar.

Todas as áreas doadoras cicatrizaram sem complicações. Não houve relato de infecção, deiscência, comprometimento do retorno venoso ou do nervo interósseo posterior. Em quatro casos, o retalho da IAP revelou-se insuficiente para cobrir completamente a falha, sendo necessário complementar com enxerto cutâneo em áreas sem exposição de tendões ou estruturas neurovasculares. No que se refere ao fechamento primário ou enxertia da área doadora, em 10 (55,5%) casos, foi necessário recorrer a enxerto.

## Discussão

Cheema et al. 10 relataram sua experiência com 64 retalhos, dos quais 88,24% sobreviveram completamente. Os quatro que falharam em seu estudo apresentaram esqueletização do pedículo. Büchler e Frey 11 relataram 4 perdas parciais em 16 casos, todos por infecção do sítio operatório. Lee et al. 12 relataram 2 perdas em 49 casos, ambas por congestão venosa.

Neste estudo, 16 pacientes tiveram boa recuperação, ao passo que 1 apresentou necrose total, e outro, parcial do retalho. No caso de necrose total, houve lesão do pedículo no momento da dissecção; já no caso de perda parcial, estima-se que o desenho do retalho foi excessivamente proximal, devido à necessidade de um longo arco de rotação.

Dessa forma, é possível estimar que o retalho é seguro para falhas da primeira comissura e, principalmente, para o dorso da mão. Caso haja necessidade de rotação do retalho em regiões distais à articulação metacarpofalangeana dos dedos longos ou além da primeira comissura, em falhas mais volares, o referido retalho pode não ser a melhor opção, pelo risco de perda. Porém, Wu et al. 13 descreveram 8 casos de cobertura de falhas em dedos longos com sucesso.

**Fig. 8:**
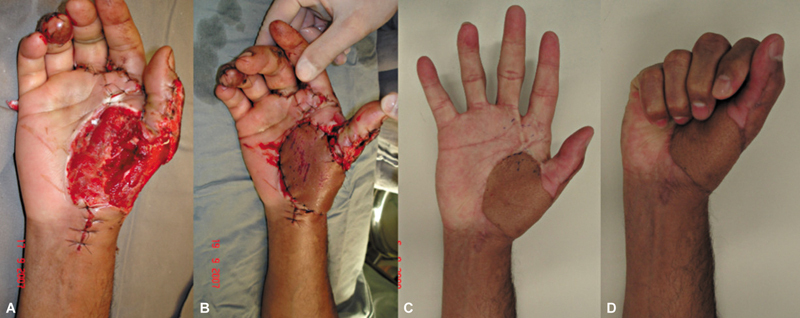
( **A** ) falha inicial; ( **B** ) pós-operatório imediato de cobertura; e ( **C,D** ) funcionalidade com 11 meses de pós-operatório.

A região volar da mão com a melhor capacidade de cobertura pelo retalho foi a região tenar, na qual, em um dos casos, conseguiu-se boa cobertura da região receptora e bom prognóstico funcional ( Fig. 8 ). Áreas da palma da mão além desse limite não são recomendadas pelos autores para cobertura.

**Fig. 9:**
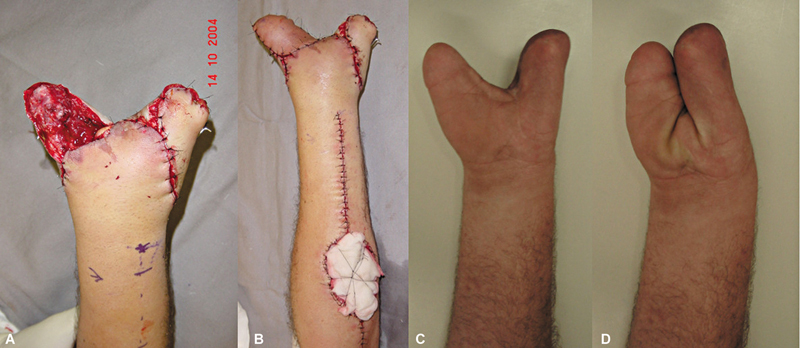
( **A** ) Falha inicial; ( **B** ) pós-operatório imediato de cobertura; ( **C,D** ) funcionalidade com 3 anos de pós-operatório.

O retalho foi essencial para o prognóstico funcional do membro também em casos de lesões mutilantes da mão. Em um caso de perda complexa de estruturas da mão, o retalho foi realizado para cobertura óssea de um raio que permitiu movimento de pinça ao paciente ( Fig. 9 ).

**Fig. 10:**
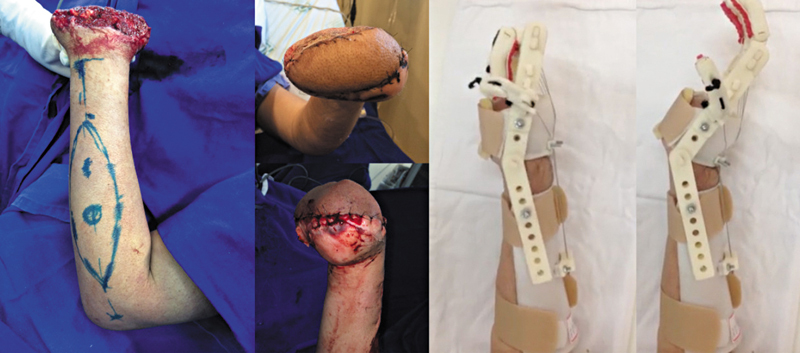
Retalho utilizado para auxiliar na protetização em amputação complexa da mão.

Em um caso grave de amputação traumática bilateral no nível do carpo, o retalho foi utilizado para cobertura mais robusta do carpo bilateral, como estágio inicial para permitir protetização mais adequada no futuro, fator que influencia também diretamente na funcionalidade do membro afetado ( Fig. 10 ).

A literatura mostra grandes variações de tamanhos no retalho. Zhou et al. 14 usaram tamanhos distintos (maior: 11 × 8,5 cm; menor: 5,3 × 2,6 cm). Na série de Kumaraswamy et al., 15 o maior retalho descrito foi de 10 × 4 cm. Mago 16 descreveu uma série de 20 casos, com 2 casos de perda total, e tamanhos que variaram de 4 × 4 cm a 10 × 8 cm. Balakrishnan et al. 17 usaram o menor retalho, de 5 × 2,5 cm, e o maior, de 21 × 10 cm. Ren et al. 8 consideraram a largura do retalho como o maior fator limitante para o seu uso, com risco de perda em retalhos maiores de 6 cm; os autores obtiveram fechamento primário em 16,7% dos casos de uma amostra de 30 pacientes. No presente trabalho, houve 1 caso que ultrapassou 6 cm de largura, com 7 cm, sem perda. Porém, é importante ressaltar que valores absolutos de medida não levam em consideração as dimensões do antebraço de cada paciente, que variam de acordo com o biotipo corporal.

Conforme relatado por vários autores, a dissecção deve começar distalmente, e os cirurgiões devem procurar a anastomose da perfurante entre artéria interóssea anterior e a AIP pelo menos 2 cm proximalmente à dobra dorsal do punho. Foi seguido o mesmo princípio nos pacientes deste estudo, e encontrou-se a perfurante em todos os casos.

Embora os cirurgiões devam estar cientes das variações anatômicas nos vasos, poucos autores relataram suas experiências quanto às variações. Durante sua dissecção, Penteado et al. 4 não encontraram a artéria interóssea anterior além do terço médio do antebraço em quatro casos, e nenhuma perfurante anastomótica foi encontrada em um caso. Em 2 casos de 36, Büchler e Frey 11 não encontraram a AIP além do terço médio do antebraço. Neste estudo, encontrou-se apenas uma variação anatômica (pedículo duplicado), que não afetou negativamente a dissecção nem o desfecho do caso.

A desvantagem do retalho da AIP é a dificuldade técnica de sua dissecção, devido ao seu pedículo fino e delicado. Além disso, requer identificação e neurólise do nervo interósseo posterior para evitar déficit de extensão de dedos. Como vantagem, esse retalho apresenta boa aceitação estética, pois a espessura da pele dorsal do antebraço é semelhante à da pele dorsal da mão. Todos os pacientes do estudo ficaram satisfeitos com o resultado cosmético.

Há limitações no estudo, como a amostra pequena e seu caráter retrospectivo, que não permite conclusões definitivas, ao contrário de estudos com maior nível de evidência. Porém, foi possível estimar parâmetros que auxiliam a técnica cirúrgica e atestam a melhor escolha de áreas receptoras para o retalho, que permitem reduzir as possibilidades de falha.

## Conclusão

O retalho da AIP é uma opção segura, previsível e versátil para a cobertura de defeitos da mão e das regiões distais do antebraço, especialmente no dorso, na primeira comissura e no polegar. A morbidade é menor quando comparada a de outros retalhos locais e microcirúrgicos, e resulta em menor tempo cirúrgico e de internação, reabilitação precoce e boa aceitação estética pelos pacientes, com bons resultados pós-operatórios.
